# Environmental and intracellular regulation of *Francisella tularensis ripA*

**DOI:** 10.1186/1471-2180-9-216

**Published:** 2009-10-12

**Authors:** James R Fuller, Todd M Kijek, Sharon Taft-Benz, Thomas H Kawula

**Affiliations:** 1 Department of Microbiology and Immunology, School of Medicine, University of North Carolina at Chapel Hill, Chapel Hill, NC, USA

## Abstract

**Background:**

*Francisella tularensis *is a highly virulent, facultative intracellular pathogen and the etiologic agent of the zoonotic disease Tularemia. RipA is a cytoplasmic membrane protein that is conserved among *Francisella *species and is required for intracellular growth. *F. tularensis ripA *deletion mutants escape the phagosome of infected cells, but unlike wild type organisms fail to replicate in the host cell cytoplasm.

**Results:**

Further analysis of *ripA *with respect to environmental effects on the growth of mutant strains and expression levels revealed that RipA is required for optimal growth at pH 7.5 but not pH 6.5. Using a combination of RT-PCR, *ripA-lacZ *transcriptional and translational fusions, and a RipA-tetracysteine tag fusion protein we found that both *ripA *transcription and RipA protein levels were elevated in organisms grown at pH 7.5 as compared to organisms grown at pH 5.5. A number of genes, including *iglA*, that are required for intracellular growth are regulated by the transcriptional regulators MglA and SspA, and are induced upon infection of host cells. We quantified *ripA *and *iglA *expression at different stages of intracellular growth and found that the expression of each increased between 1 and 6 hours post infection. Given the similar intracellular expression patterns of *ripA *and *iglA *and that MglA and SspA are positive regulators of *iglA *we tested the impact of *mglA *and *sspA *deletions on *ripA *and *iglA *expression. In the deletion mutant strains *iglA *expression was reduced dramatically as expected, however *ripA *expression was increased over 2-fold.

**Conclusion:**

Expression of *ripA *is required for growth at neutral pH, is pH sensitive, and is responsive to the intracellular environment. The intracellular expression pattern of *ripA *coincided with *iglA*, which is positively regulated by MglA and SspA. However, in contrast to their positive impact on *iglA *expression, MglA and SspA negatively impacted *ripA *expression *in vitro*.

## Background

*Francisella tularensis *is a highly virulent Gram negative bacterial pathogen and the etiologic agent of the zoonotic disease tularemia. The bacteria are spread via multiple transmission routes including arthropod bites [1], physical contact with infected animal tissues [2], contaminated water [3,4], and inhalation of aerosolized organisms [5]. Inhalation of as few as 10 colony forming units (CFU) are sufficient to initiate lung colonization [6,7] and the subsequent development of pulmonary tularemia, which is the most lethal form of the disease exhibiting mortality rates as high as 60% [8].

*F. tularensis *is a facultative intracellular pathogen that invades, survives and replicates within numerous cell types including, but not limited to, macrophages [9,10], dendritic cells [11], and alveolar epithelial cells [12]. Intracellular growth is intricately associated with *F. tularensis *virulence and pathogenesis, and the intracellular lifestyle of *F. tularensis *is an active area of investigation. Following uptake or invasion of a host cell wild type *F. tularensis *cells escape the phagosome and replicate within the cytoplasm [13-15] of infected cells. The phagosome escape mechanism employed by *F. tularensis *remains essentially unknown, but this property is clearly necessary for *F. tularensis *intracellular growth since mutants that fail to reach the cytoplasm are essentially unable to replicate within host cells [16,17].

Following phagosome escape *F. tularensis *must adapt to the cytoplasmic environment. Purine auxotrophs [18], acid phosphatase [19], *clpB *protease [20], and *ripA *mutants [21] reach the cytoplasm but are defective for intracellular growth. RipA is a cytoplasmic membrane protein of unknown function that is conserved among *Francisella *species [21].

Notably, the majority of attenuating mutations described to date impart intracellular growth defects on the mutant strains. We recently identified a locus, *ripA*, that encoded a cytoplasmic membrane protein that was conserved among *Francisella *species. Mutant strains lacking *ripA *entered host cells and escaped the phagosome, but were defective for intracellular growth [21]. The deletion mutants had no apparent affect on *F. tularensis *growth with respect to doubling time or final density when propagated in Chamberlains chemically defined media or complex nutrient rich BHI. Thus, expression of *ripA *appeared to be required for adaptation and growth in the cytoplasmic environment of a host cell.

The expression of a number of *Francisella *virulence factors required for phagosomal escape and intracellular replication are induced in the intracellular environment by a process involving the positive transcriptional regulators MglA and SspA [16,22-24]. Data on whether MglA regulates *ripA *expression is contradictory. Microarray analysis of MglA regulated loci indicated that *ripA *expression was unaffected by MglA, [23], whereas results from a proteomics study suggested that RipA was repressed by MglA [25].

Given the *ripA *deletion mutant phenotype with respect to intracellular growth, that MglA and SspA regulate numerous genes required for intracellular growth and that there is a discrepancy between the microarray and proteomic results with respect to MglA affects on *ripA *expression, we applied multiple approaches to investigate environmental requirements for, and influences on, *F. tularensis ripA *expression.

## Results

### Characterization of the *ripA *locus and transcriptional unit

**Figure 1:**
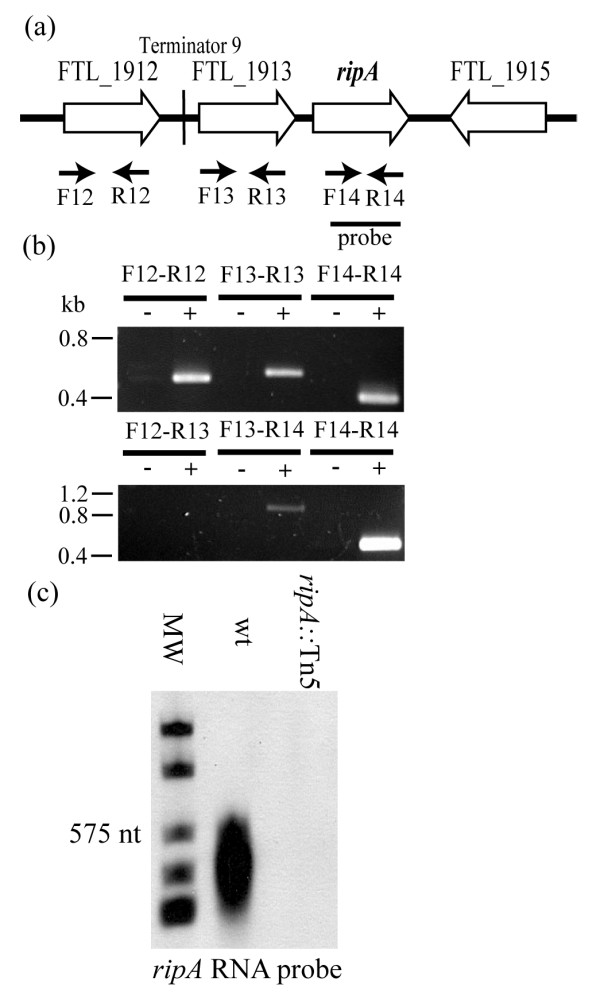
**The *ripA *genomic region and transcript analysis**. (a) Graphical representation of the *F. tularensis *LVS *ripA *genomic region. Primers utilized for RT-PCR are marked with arrows while the region complementary to the RNA probe used in the Northern analysis is demarcated by a solid line. (b) RT-PCR analysis of the expression of genes FTL_1912 (F12-R12), FTL_1913 (F13-R13), and *ripA *(F14-R14) are shown in the upper image. Analysis for transcripts bridging FTL_1912 to FTL_1913 (F12-R13) and FTL_1913 to *ripA *(F13-R14) shown in lower image and compared to the intrageneic *ripA *amplicon (F14-R14). PCR of cDNA demarcated by a (+) and reverse transcriptase negative reactions to assess DNA contamination marked as (-). (c) Northern analysis to evaluate the transcript size of *ripA *containing RNA. Roche digoxigenin labeled RNA ladder is present in the left most lane followed by total RNA from *F. tularensis *LVS (wt) and *F. tularensis *LVS *ripA*:: Tn5. This analysis used a *ripA *complementary digoxigenin labeled RNA probe demonstrating the presence of monocistonic *ripA *transcript in LVS and the absence of the transcript in *F. tularensis *LVS *ripA::*Tn5.

Prior to analyzing *ripA *expression patterns and regulation we sought to determine the context and extent of the *ripA *locus and transcript, respectively. The genome annotation suggests that the gene following *ripA*, FTL_1915, would be transcribed in the opposite orientation (Fig 1a). Preceding *ripA *are two genes, FTL_1912 and FTL_1913 that are predicted to be transcribed in the same orientation, and thus could constitute a three gene operon. We tested this possibility by RT-PCR and Northern blot analysis.

Individual primer sets were designed to amplify coding regions from each of the three genes, and another set was designed to amplify any RNA transcripts that bridged adjacent genes (Fig. 1a). Twenty ng of synthesized first strand cDNA was subjected to 25 or 30 cycles of amplification to synthesize intragenic and potential gene bridging (intergenic) products, respectively. There was no detectible product following amplification with primers bridging FTL_1912 and FTL_1913 (Fig. 1b), suggesting that a predicted Rho independent terminator located between the two genes was functional. A faint amplification product was present in reactions using FTL_1913 - *ripA *bridging primers (Fig. 1b). However, the band intensity was significantly lower than that of the *ripA *amplicon and was detectable only after the additional cycles of amplification. This result suggests that the FTL_1913 transcript terminates, albeit less efficiently than that of FTL_1912, and that *ripA *expression was initiated independently from the FTL_1912 promoter.

Total RNA harvested from mid exponential phase *F. tularensis *LVS and *F. tularensis *LVS *ripA::*Tn5(Table 1) was evaluated by Northern blot analysis to determine the *ripA *transcript size. The *ripA *coding sequence is 537 nucleotides, and an approximately 600 nucleotide RNA fragment hybridized to an anti-sense *ripA *probe confirming that the *ripA *gene was transcribed (Fig. 1c), and supporting the RT-PCR data that potential co-expression with FTL_1913 is negligible, at best. No *ripA *message was detected in the *F. tularensis *LVS *ripA::*Tn5 RNA samples demonstrating the specificity of the *ripA *probe.

**Table 1:** Bacterial strains and plasmids.

*Strains or Plasmid*	*Description*	*Source or Reference*
**Bacteria**		
*Francisella tularensis *LVS	*F. tularensis *live vaccine strain	CDC, Atlanta, GA
*ripA*::Tn5	Tn5 *ripA *transposon mutant	[21]
*ripA*::pBSK Φ(*ripA'-lacZ*)*1*	Plasmid cointegrate	This work
*ripA*:: pBSK Φ(*ripA'-lacZ*)*2*	Plasmid cointegrate	This work
*iglA*:: pBSK Φ(*iglA'-lacZ*)	Plasmid cointegrate	This work
Φ(*ripA'-TC*)	Exchanged allele	This work
*ΔmglA*	Inframe deletion of *mglA*	This work
*ΔmglA ripA*:: pBSK Φ(*ripA'-lacZ*)*2*	Plasmid cointegrate	This work
*ΔmglA iglA*:: pBSK Φ(*iglA'-lacZ*)	Plasmid cointegrate	This work
*ΔsspA*	Inframe deletion of *sspA*	This work
*ΔsspA ripA*:: pBSK Φ(*ripA'-lacZ*)*2*	Plasmid cointegrate	This work
*ΔsspA iglA*:: pBSK Φ(*iglA'-lacZ*)	Plasmid cointegrate	This work
*ΔmglA ΔsspA*	Inframe gene deletions	This work
*ΔmglA ΔsspA ripA*:: pBSK Φ(*ripA'-lacZ*)*2*	Plasmid cointegrate	This work
*ΔmglA ΔsspA iglA*:: pBSK Φ(*iglA'-lacZ*)	Plasmid cointegrate	This work
**Plasmids**		
pBSK *bla lacZ*	pBluescript cloning vector	Stratagene
pBSK *lacZ aphA1 bla*	Transcriptional *lacZ *fusion	This work
pBSK *lacZ cat bla*	Translational *lacZ *fusion	This work
pBSK Φ(*ripA'-lacZ*)*2 aphA1 bla*	*Francisella *suicide vector	This work
pBSK Φ(*ripA'-lacZ*)*1 cat*	*Francisella *suicide vector	This work
pBSK Φ(*iglA'-lacZ*)*2 aphA1*	*Francisella *suicide vector	This work
pMP590	*Francisella sacB *suicide vector	[47]
pMP590 *ΔmglA*	*mglA *allelic exchange vector	This work
pMP590 *ΔsspA*	*sspA *allelic exchange vector	This work
pMP590 Φ(*ripA'-TC*)	Φ(*ripA'-TC*) suicide vector	This work
pMP633	*Francisella *shuttle vector	[47]
pMP633 *mglA*^+^	*mglA*^+ ^with native promoter	This work
pMP633 *sspA*^+^	*sspA*^+ ^with native promoter	This work
pKK MCS	*Francisella *shuttle vector	[21]
pKK MCS Φ(*ripA'-lacZ*)*1*	translational fusion	This work
pKK MCS Φ(*ripA'-lacZ*)*1a*	-10 mutation	This work
pKK MCS Φ(*ripA'-lacZ*)*1b*	RBS mutation	This work
pKK MCS Φ(*ripA'-lacZ*)*1c*	*lacZ *frameshift	This work
pKK MCS Φ(*ripA'-lacZ*)*1d*	*ripA *core promoter	This work
pKK MCS Φ(*ripA'-lacZ*)*2*	transcriptional fusion	This work
pKK MCS Φ(*ripA'-TC*)	*ripA*-CT tetracysteine tag fusion	This work

### Quantifying *ripA *expression with transcriptional and translational *lacZ *fusions

**Figure 2:**
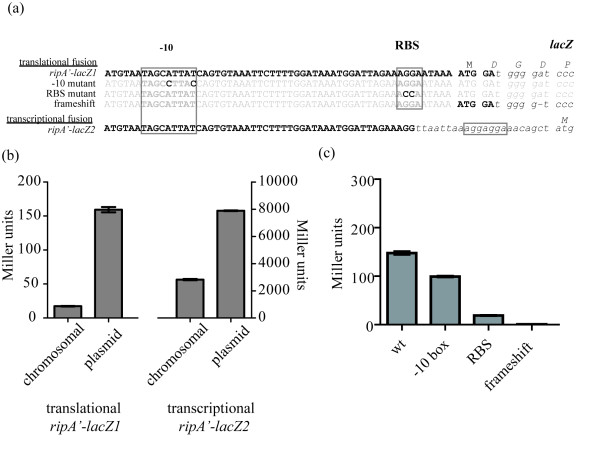
**The *ripA'-lacZ *reporter sequence and expression**. (a) Multiple sequence alignment of translational and transcriptional *ripA'-lacZ *fusions. Predicted -10 and RBS sequences are boxed with introduced mutations in each highlighted. (b) β-galactosidase activity of chromosomal and plasmid translational and transcriptional *F. tularensis *LVS *ripA'-lacZ *reporter strains displayed as mean Miller units. Error bars represent the standard deviation of three samples. (c) β-galactosidase activity of *F. tularensis *LVS plasmid translational *ripA'-lacZ1 *promoter mutations displayed as mean Miller units. Error bars represent the standard deviation of three samples.

To facilitate studies on *ripA *expression patterns and properties we constructed transcriptional and translational *ripA-lacZ *fusion strains (Table 1) so that β-galactosidase assays could be used to conveniently quantify *ripA *expression under a multitude of conditions. The translational *ripA'-lacZ1 *was created by cloning the entire *ripA *5' untranslated region from the end of the previous gene through the *ripA *start codon plus 2 additional bases in-frame with *lacZ *beginning at the second *lacZ *codon (Fig. 2a). The transcriptional *ripA'-lacZ2 *fusion was constructed by cloning the same 5' untranslated region of *ripA *minus the 6 bases immediately preceding the start codon to the complete *lacZ *gene including the *lacZ *ribosome binding site (Fig. 2a). These two constructs were cloned into pKK MCS and transformed into *F. tularensis *LVS creating plasmid based reporter strains (Table 1).

**Figure 3:**
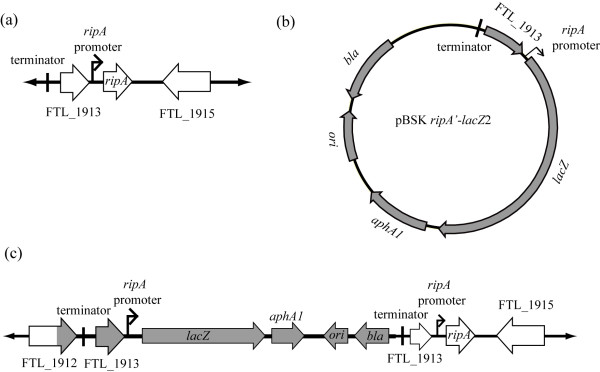
**Reporter plasmids and co-integrates**. Cartoon representations of the *F. tularensis *LVS genomic organizations of the *ripA *locus (a), pBSK *ripA'-lacZ*2 transcriptional reporter plasmid (b), and the *ripA*::pBSK *ripA'lacZ *cointegrate (c). The *ripA *locus is present in only one copy in *ripA*::pBSK *ripA'-lacZ*2 however the promoter is duplicated by the insertion resulting maintenance of the entire wild type *ripA *locus as well as the *ripA'-lacZ *reporter. The predicted *ripA *promoter is represented by a black arrow (a-c). pBSK *ripA'-lacZ*2 is shown in gray while the alleles of the native locus are white.

The transcriptional and translational fusion constructs were also cloned into pBSK (Table 1), which cannot replicate in *Francisella*, and integrated into the LVS chromosome via single cross over recombination creating LVS *ripA*::pBSK *ripA'-lacZ*2 and LVS *ripA*::pBSK *ripA'-lacZ1*, respectively. The integration of the fusion constructs into the wild type *ripA *locus resulted in both *ripA*^+ ^(Fig. 3a) and *ripA'-lacZ *fusion alleles (Fig. 3b) on the chromosome (Fig. 3c). The insertions did not impact intracellular replication of the reporter strains and thus were unlikely to significantly impact expression of the wild type *ripA *gene.

We examined the effects of specific mutations in the predicted *ripA *promoter, ribosome binding site, and translation frame on the expression of β-galactosidase. Mutations in the predicted -10 sequence, RBS, and the introduction of a frameshift mutation (Fig. 2a) in the translational fusion construct each resulted in decreased β-galactosidase activity as compared to the wild type reporter (Fig. 2c).

The β-galactosidase activity expressed by the chromosomal reporters was less than 25% of that produced by the plasmid reporters (Fig. 2b). The *ripA'-lacZ*1 translational fusion produced significantly less activity than the *ripA'-lacZ*2 transcriptional fusion in both the chromosomal and plasmid version of the reporter (Fig. 2b). These differences might reflect post transcriptional regulation of expression or simply a difference in the efficiency of translation initiation between the two constructs.

### Quantification of RipA protein

**Figure 4:**
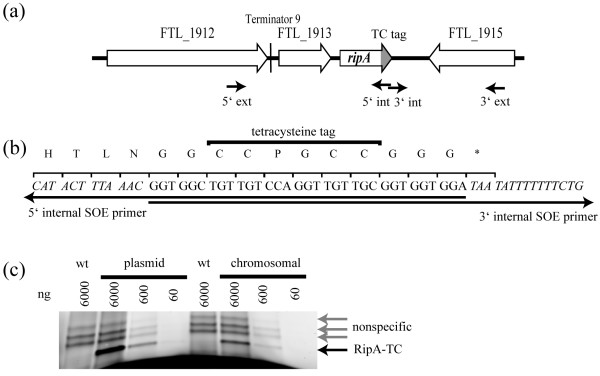
**Tetracysteine tag construction and expression**. (a) Graphical depiction of *F. tularensis *LVS *ripA *locus showing the location of SOE PCR primers used to insert the C terminal TC tag (marked in gray). (b) Nucleotide and amino acid sequence of the C terminal TCtag showing the overlapping sequence of the SOE PCR primers. (c) In gel fluorescence of RipA-TC (black arrow) from dilution series of *F. tularensis *LVS (plasmid) pKK *ripA'-TC *and *F. tularensis *LVS (chromosomal)*ripA'-TC *using 6000 ng to 60 ng total protein of whole cell lysates. *F. tularensis *LVS lysates (wt) used as a non TC tagged control displaying three non specific bands (gray arrows) at a higher molecular weight than RipA-TC.

We were unable to quantify native RipA protein concentrations in *Francisella *cultures since our polyclonal anti-RipA antisera produced high background in Western blots and ELISA [21]. We therefore generated a construct that expressed a RipA - tetracysteine (TC) fusion protein to facilitate the use of FlAsH™ (Invitrogen) reagents to directly measure RipA protein concentrations. Both plasmid and chromosomal integrant strains (Fig. 4a) expressing RipA-TC (Fig. 4b) were constructed in a *ΔripA *background. Intracellular replication was restored in each of these strains demonstrating that the RipA-TC fusion protein was functional and did not confer a detectable mutant phenotype (data not shown).

Whole cell lysates prepared from mid exponential phase bacteria growing in Chamberlains defined media were suspended in FlAsH™ loading buffer containing biarsenical fluorescein and subjected to SDS-PAGE. The RipA-TC fusion protein was detected and quantified by relative mean fluorescence with wild type *F. tularensis *LVS lacking any TC fusion protein serving as a control to identify background and non-specific fluorescence. To determine the detection limits of the TC tag fusion protein assay, whole cell lysates (6000 ng to 60 ng total protein) of LVS expressing chromosomal (Fig. 4a) or plasmid *ripA'-TC *fusion alleles were incubated with FlAsH™ reagent, separated via SDS-PAGE and subjected to in - gel fluorescence measurement. There were 3 nonspecific biarsenical fluorescein binding proteins between 22 kDa and 30 kDa in size in wild type *F. tularensis *LVS lysates, which were easily distinguishable from RipA-TC which migrated at approximately 18 kDa (Fig. 4c). RipA-TC expressed from plasmid was detectable in the 60 ng whole cell lysate samples whereas chromosomally expressed was detected in 600 ng samples (Fig. 4c). The concentration of RipA-TC (plasmid) was approximately 6.5 fold greater than RipA-TC (chromosome). Thus, the use of the RipA-TC fusion in conjunction with biarsenical labeling provided a sensitive and reproducible method to detect and quantify RipA in *Francisella*.

### Expression of *ripA *is affected by pH

**Figure 5:**
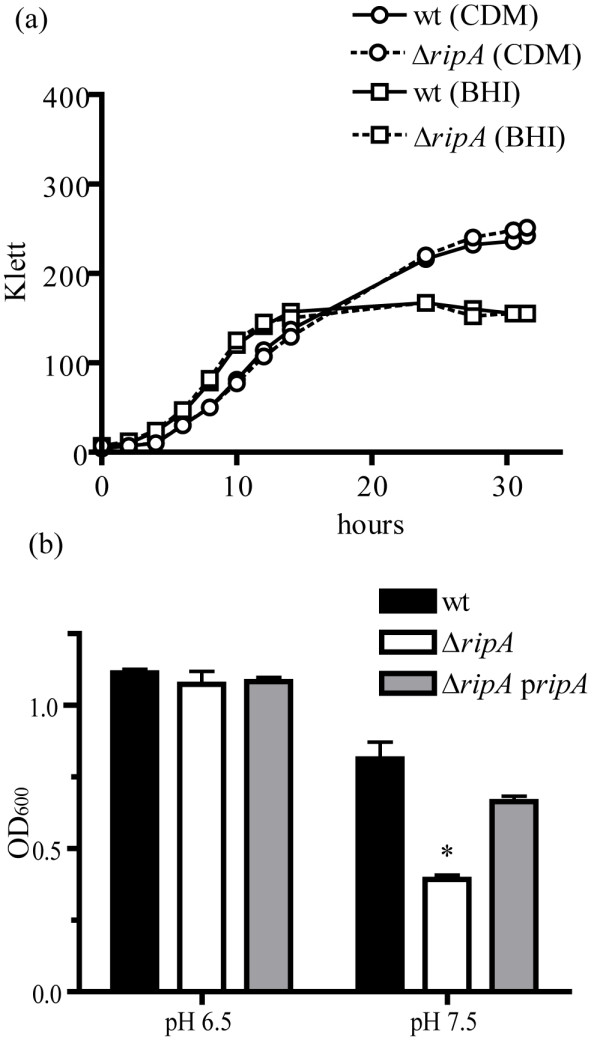
**Analysis of pH effects on growth**. (a) Effect of pH and media on the growth of *F. tularensis *LVS wild type (wt) and *ΔripA *strains. The initial pH of BHI and CDM was measured as 7.3 and 6.3 respectively. Cultures were seeded at time zero with 1.12 × 10^8 ^CFU/ml. Klett measurements were recorded at the listed times. The growth curves displayed are a representative example of growth under the indicated conditions. *F. tularensis *growth over time shifts the pH of the media by the secretion of ammonia. The initial pH of the media shifts by < 0.2 pH units by 6 hours and from 0.5 to 1.0 pH units by 24 hours. (b) The growth of *F. tularensis *LVS (wt), Δ*ripA*, and Δ*ripA *p*ripA *in CDM with a starting pH of 6.5 or 7.5 was measured at 24 hours. The mean OD_600 _of four replicates is represented with error bars representing ± one standard deviation. The growth of *F. tularensis *LVS Δ*ripA *was significantly less (P < 0.05) than wild type and the Δ*ripA *p*ripA *strain as tested using a Student's t test.

We previously reported that *F. tularensis *LVS Δ*ripA *had no discernable growth defects in CDM [21]. While evaluating the characteristics of a Δ*ripA *strain in a variety of environmental conditions we found that the growth of the mutant was pH sensitive. The reported optimal pH for the growth of *F. tularensis *in CDM is 6.2 to 6.4 [26]. *F. tularensis *LVS *ΔripA *grew at the same rate and extent as wild type at this pH (Fig. 5a). However, when the initial pH of CDM was set to 7.5 the mutant achieved maximum densities significantly lower than that of wild type *F. tularensis *LVS (P < 0.05, Fig. 5b). In 4 independent tests the mean OD_600 _achieved by *F. tularensis *LVS Δ*ripA *grown for 24 hours in CDM with an initial pH of 7.5 was 0.448 ± 0.06 versus 0.732 ± 0.2 for wild type LVS (P < 0.05). This is an intriguing result since the described pH of the macrophage cytoplasm is approximately 7.4 [27] and *F. tularensis *LVS *ΔripA *fails to replicate in the cytoplasm [21]. This growth defect was not evident when the mutant was cultivated in the complex rich media BHI (Fig. 5a), which had an initial pH of approximately 7.3. Minimal media and neutral pH were both necessary for the growth defect. Thus, the defect may be due to the effects of pH on nutrient acquisition in the mutant.

**Figure 6:**
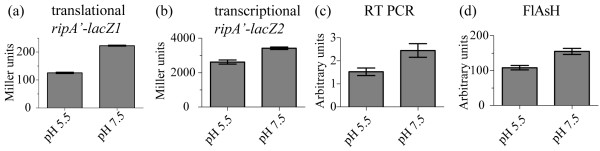
**Analysis of the effects of pH on expression**. Effect of pH on *F. tularensis *LVS *ripA *expression. All experiments were performed using mid exponential phase bacteria cultured in Chamberlains defined media at pH 5.5 or pH 7.5. Data are presented as mean values with error bars representing one standard deviation. (a) β-galactosidase activity of *F. tularensis *LVS pKK *ripA'-lacZ1 *at pH 5.5 and pH 7.5. Difference in expression levels were significant (P < 0.01). (b) β-galactosidase activity of *F. tularensis *LVS *ripA'-lacZ2 *at pH 5.5 and pH 7.5. Difference in expression levels were significant (P < 0.01). (c) *F. tularensis *LVS *ripA *RNA concentrations displayed as *tul4 *normalized mean trace (Int mm) on four independent RT-PCR reactions using purified total RNA samples of mid exponential *F. tularensis *LVS cultured at pH 5.5 and pH 7.5. Difference in expression levels were significant (P < 0.01). (d) RipA-TC concentration in whole cell lysates of mid exponential phase *F. tularensis *LVS *ripA'-TC *cultured at pH 5.5 and pH 7.5. Concentrations were measured using densitometry of the specific in-gel fluorescence of FlAsH™ labeled RipA-TC. Four independent samples were used to calculate mean expression. Difference in expression was significant (P < 0.01).

We hypothesized that conditions under which *ripA *was necessary for growth might also impact *ripA *expression. We therefore used the *ripA-lacZ *fusion strains to examine the effects of pH on *ripA *expression. β-galactosidase activity was measured from mid-exponential phase cultures grown in Chamberlains defined media at pH 5.5 and 7.5, at which time the media was within 0.2 units of the initial pH. The plasmid-encoded translational reporter strain expressed 125 ± 3 and 223 ± 2 Miller units at pH 5.5 and 7.5, respectively (Fig. 6a) representing a 1.8 fold difference (P < 0.001). The chromosomal transcriptionreporter strain expressed 2618 ± 121 and 3419 ± 71 Miller units at pH 5.5 and 7.5, respectively (Fig. 6b) representing a 1.3 fold (P = 0.0016).

RT-PCR and FlAsH™ labeling of RipA-TC were used as complementary assays for comparison to the *lacZ *fusion results. The *ripA *transcript levels were evaluated by RT-PCR in replicates of four independent cultures and normalized to *tul4 *[22]. Primers internal to *ripA *and *tul4 *were designed with matched melting temperatures and amplification product sizes. Total RNA was collected from *F. tularensis *LVS cultures at mid exponential stage growing in Chamberlains defined media at pH 5.5 and pH 7.5. cDNA was generated from the RNA samples using random primers in a reverse transcriptase reaction. Samples lacking reverse transcriptase were used to monitor DNA contamination. Quantization of *ripA *transcripts was achieved by densitometry of gene-specific products isolated by agarose electrophoresis. Mean normalized expression of *ripA *± standard deviation at pH 5.5 was 1.527 ± 0.1656 and 2.448 ± 0.2934 at pH 7.5 (Fig. 6c) representing a 1.6 fold expression differential (P = 0.0033).

The concentration of RipA protein present at pH 5.5 and pH 7.5 was measured by FlAsH™ labeling of RipA-TC present in whole cell lysates of the chromosomal fusion strain (Table 1). Six μg of total protein was incubated with TC specific FlAsH™ reagents, separated by SDS-PAGE and subjected to in-gel fluorescence. Mean intensity of RipA-TC ± standard deviation of four independent samples at pH 5.5 was 1.083 × 10^7 ^± 6.340 × 10^5 ^arbitrary units as compared to 1.551 × 10^7 ^± 8.734 × 10^5 ^arbitrary units at pH 7.5 (Fig. 6d), representing a 1.43 fold change in expression (P = 0.00031) as compared to the 1.8 fold difference expressed by the *ripA'-lacZ1 *translational fusion. Results from the four different measures of *ripA *expression revealed pH - affected increases ranging from 1.3 to 1.8 fold. While the increased *ripA *expression at pH 7.5 as compared to 5.5 is mathematically statistically significant, it remains to be seen if is biologically relevant.

### *F. tularensis *LVS *ripA *expression during intracellular growth

**Figure 7:**
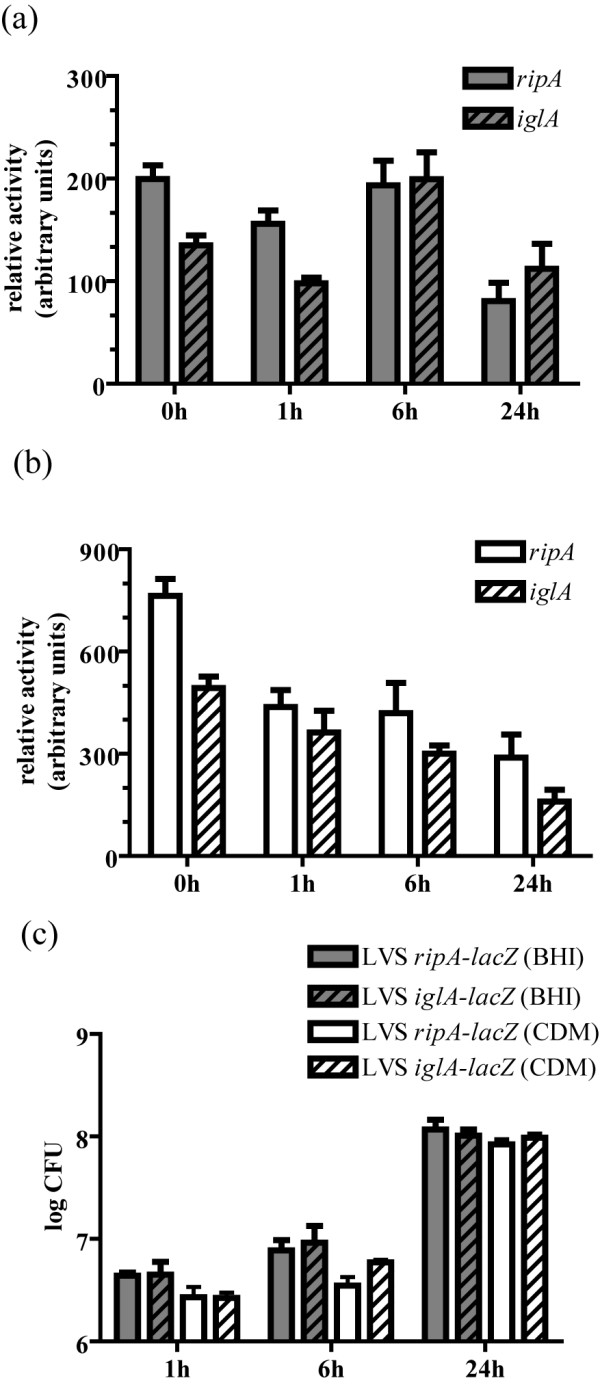
**Expression of *ripA *in the intracellular niche**. Intracellular expression of LVS *ripA'-lacZ2 *and LVS *iglA'-lacZ *in J774A.1 mouse macrophage like cells infected at an MOI of 100. Inoculums were either prepared from mid exponential phase bacteria grown in BHI (a) or CDM (b) as indicated in the legend. Preparation in CDM resulted in an increased initial activity in the reporter strains. All assays were performed on four replicate wells and reported as mean relative activity ± standard deviation. Inoculums activity was calculated from four samples taken before application of the inoculums. Mean β-galactosidase activity is normalized by time of development and CFU per well minus the activity from the control samples. All differences in expression were significant (P < 0.05) with the exception of comparisons between *ripA'-lacZ2 *inoculums to 6 h, and *iglA'-lacZ *1 h to 24 h. The mean CFU recovered at each time point assayed are displayed as log CFU (c). Error bars represent the standard deviation of four samples. Each strain invaded and replicated by 24 hours in J774A.1 mouse macrophage like cells.

The pH effect on *ripA *expression parallels the location-specific requirement for functional RipA within the host cell. That is, RipA is dispensable for the early stages of invasion and phagosome escape where the pH is likely to be relatively acidic, but is required for replication in the more neutral pH of the cytoplasm, a condition where *ripA *expression is elevated. To see if this correlation exists throughout the course of infection we measured β-galactosidase produced by the *F. tularensis *LVS chromosomal transcriptional *ripA-lacZ2 *fusion strain at different stages of intracellular growth. Since the *iglA *gene is induced during intracellular growth [28], we therefore constructed and used an *iglA-lacZ *transcriptional reporter for control and comparison purposes. The *iglA-lacZ *fusion was cloned into pBSK *aphA1 *(Table 1) and integrated into the *F. tularensis *LVS chromosome as described earlier for *ripA*. The insertion of pBSK *iglA'-lacZ *into the chromosome likely has polar effects on *iglB*, *iglC*, and *iglD*. However, since this operon is on the Pathogenecity Island which is duplicated in *F. tularensis *LVS this reporter construct strain still has an intact *igl *locus. We cannot say definitively that this reporter strain has no deficiencies, but there were no detectable differences between this strain and wild type *F. tularensis *LVS with respect to intracellular replication rate or extent (Fig 7c).

We predicted that the conditions under which the cultures were prepared might affect the *ripA *and *iglA *expression levels prior and subsequent to internalization by host cells. Therefore, the activities of *ripA'-lacZ*2 and *iglA'-lacZ *transcriptional fusions were measured from cultures grown in BHI and CDM to assess the impact of complex nutrient rich and chemically defined minimal media, respectively, on their expression. The mean activity of each reporter was *ca*. 1.6 fold higher in CDM relative to BHI (P < 0.01) (Fig 7ab). Given the effect of growth media on *ripA *and *iglA *we measured and compared the expression of these genes in cells infected with the reporter strains propagated in each of these media.

To initiate the intracellular expression analyses host cell entry was synchronized by centrifugation of reporter strains onto chilled J774A.1 monolayers as described [29]. β-galactosidase activity was measured in the inoculums, and at 1, 6, and 24 hours post inoculation using a modified β-galactosidase assay similar in concept to the Miller assay but based on the rate and amount of CPRG conversion per CFU.

The mean β-galactosidase activity (± standard deviation) of *F. tularensis *LVS *ripA'-lacZ2 *at 0 (inoculum), 1, 6, and 24 hours post infection when the inoculum was prepared from BHI cultures was 199.7 (± 13.32), 155.9 (± 12.96), 193.5 (± 23.99), and 80.6 (± 17.83), respectively (Fig. 7a). The activity-galactosidase level remained similar to that of the inoculum at 1 hour post infection, increased slightly at 6 hours then dropped at 24 hours to a level that was significantly less than for all other time points (P < 0.05). When prepared in CDM the β-galactosidase levels started at a much higher value than that of the BHI-grown samples, and steadily decreased until the lowest measurement at 24 hours post inoculation (Fig. 7b).

Expression of *iglA *prepared in BHI was 135.0 (± 9.59), 97.8 (± 9.59), 199.4(± 26.24), and 112.0 (± 24.21) for the inoculum, 1, 6, and 24 hours post inoculation, respectively (Fig. 7a). The most significant change was a two fold increase at 6 hours post inoculation relative to 1 and 24 hours post inoculation (P < 0.01). By 24 hours post inoculation the relative activity returned to levels similar to that of the inoculum and at 1 hour post inoculation. The 6 hour post inoculation spike of *iglA *expression did not occur when the bacteria were prepared in CDM (Fig. 7b). As with the *ripA *fusion strain, β-galactosidase levels were significantly higher in the inoculums and throughout the course of infection. Both fusion strains invaded and replicated in the J774A.1 cells (Fig. 7c) demonstrating that the reporter integrations did not impact intracellular replication. Also, even though growth media significantly impacted *ripA *and *iglA *expression levels throughout the experiment, it had no discernable effect on host cell invasion or replication.

### The effects of *mglA *and *sspA *deletions on *ripA *expression

MglA and SspA are transcriptional regulators that associate with DNA and RNA-polymerase and modulate the expression of a number of stress response and virulence associated genes, including *iglA*, in *F. tularensis *[22-25]. In a recent study comparing protein expression profiles of wild type and *mglA *mutant strains both IglA and RipA protein levels were affected in the *mglA *mutant [25]. We investigated further the relationship between these regulators and RipA expression using the *ripA'-lacZ2 *and *iglA'-lacZ *transcriptional fusions in Δ*mglA *and Δ*sspA *mutant strains (Table 1).

**Figure 8:**
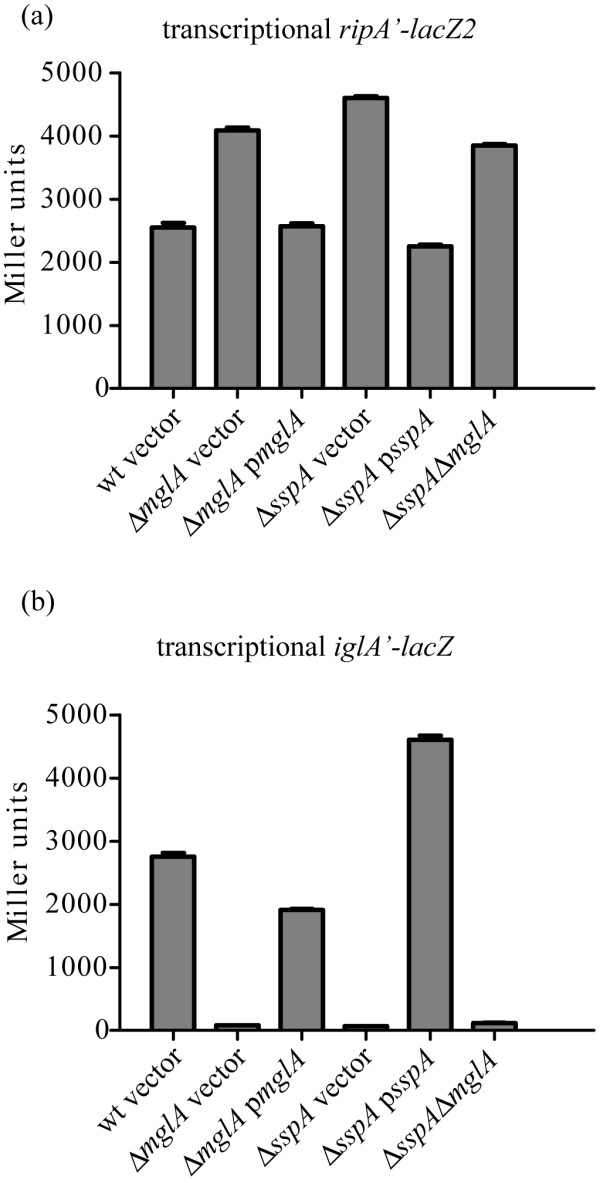
**MglA and SspA effects on *ripA *and *iglA *expression**. Mid exponential phase cultures of the indicated transcriptional *lacZ *reporter strains cultured in Chamberlains defined media were assayed for β-galactosidase activity in replicates of three and reported as mean Miller units ± standard deviation. (a) *F. tularensis *LVS *ripA'-lacZ2 *expression in wild type (wt), *ΔmglA*, *ΔsspA*, and *ΔmglAΔsspA *backgrounds. *In trans *complementation (p*mglA *and p*sspA*) was accomplished using wild type alleles and native promoters cloned into pMP633. *F. tularensis *LVS pMP633 was used as the vector only control (vector). (b) *F. tularensis *LVS *iglA'-lacZ *expression in wild type (wt), *ΔmglA*, *ΔsspA*, and *ΔmglAΔsspA *backgrounds.

β-galactosidase assays were performed on mid exponential phase reporter strains grown in Chamberlains defined media. The mean expression of *ripA *was nearly 2-fold higher (P < 0.01) in the Δ*mglA *(4091 ± 75) and Δ*sspA *(4602 ± 52) strains as compared to wild type (2549 ± 128) (Fig. 8a). Wild type levels of expression were restored by the wild type *mglA *and *sspA *alleles in the complemented mutant strains (Fig. 8a).

As expected the *mglA *and *sspA *deletions had the opposite effect on *iglA *expression. The mean expression (± standard deviation) of *F. tularensis *LVS *iglA'-lacZ *was substantially decreased in both the Δ*mglA *(80 ± 2.2) and Δ*sspA *(67 ± 0.9) strains versus wild type (2757 ± 98) (Fig. 8b). The differences of *iglA *expression in the mutant backgrounds were all significantly different from wild type (P < 0.01), and near wild type levels of expression were restored by complementation with *mglA *and *sspA in trans *(Fig. 8b). Together, these results confirm that *mglA *and *sspA *expression positively influence *iglA *expression, and conversely demonstrate that these two regulators negatively influence *ripA *expression.

## Discussion

As a facultative intracellular pathogen, *F. tularensis *is able to survive and replicate within several different types of eukaryotic cells as well as in a number of extracellular environments [9,11,12,29-32]. Other facultative intracellular pathogens such as *Salmonella typhimurium *[33], *Legionella pneumophila *[34], and *Listeria moncytogenes *[35,36] are similarly capable of adapting to multiple environments. These organisms exhibit differential gene expression in response to entering or exiting host cells, and even as they transition between intra-vacuolar and cytoplasmic niches. Mapping the gene expression profiles that accompany different stages of infection have helped to identify environmental cues that impact gene expression and virulence.

Studies on intracellular gene expression by *Francisella *species have revealed a number of genes including *iglC *[37], *iglA *[28] and *mglA *[38], that are induced upon entry and growth in macrophages. IglC protein concentrations increased between 6 hours and 24 hours post host cell invasion [37]. Similarly IglA protein concentrations increased between 8 hours and 12 hours post invasion as measured by Western blot [28]. In the current study we found that *iglA *expression was increased during intracellular growth, but only for a limited period of time. This increase in expression did not occur immediately after host cell invasion, but rather coincided with the time frame associated with the early stage of replication following phagosome escape.

We found that the laboratory growth media used to propagate the bacteria affected both *ripA *and *iglA *expression levels. Reporter activity of *ripA'-lacZ *and *iglA-lacZ *transcriptional fusions were each significantly higher in inoculums prepared in CDM vs. those prepared in BHI. As a consequence, the results of intracellular expression assays were dependent on the type of media in which the organisms were grown prior to infection. Since the initial expression levels of *ripA *and *iglA *were significantly higher in CDM grown organisms, the relative *in vivo *expression levels of these genes actually decreased throughout the course of infection. Modest increases in *iglA *and *ripA *expression during intracellular growth were observed only when organisms were propagated in BHI prior to infection. These observations are in line with that of Hazlett et. al. who found that *Francisella *virulence genes are variably expressed in different types of media, some of which more closely replicate intracellular expression profiles than others [39].

When infected with BHI-grown organisms, *F. tularensis ripA *and *iglA *gene expression changes coincided with the transitions from vacuolar, to early cytoplasmic, and then late cytoplasmic stages of infection. The expression of *ripA *was repressed during the early stage of infection when the bacteria are reportedly associated with a phagosome [13-15]. Expression of both *ripA *and *iglA *increased during the early phase of cytoplasmic growth then decreased during the latter stages of infection. The *ripA *expression levels associated with these sites and stages of intracellular growth corresponded to our observed effects of pH on *ripA *expression in CDM and the reported pH of the relevant intracellular environment. A number of studies have shown that the early *Francisella *- containing phagosome is acidified prior to bacterial escape [40,41]. Interestingly, we found that acidic pH repressed *ripA*. Additionaly, *ripA *expression was dispensable for growth at acidic pH *in vitro*, and was likewise dispensable for survival and escape from the phagosome. The pH of the cytosol of a healthy macrophage is reportedly *ca*. 7.4. Neutral to mildly basic pH resulted in increased *ripA *expression *in vitro*. The *ripA *deletion mutant was defective for growth both at neutral pH *in vitro*, and within the cytoplasm of host cells. Finally, the pH of the cytosol during late stages of *Francisella *infection has not been measured, however during apoptosis the pH reportedly drops to 5.8 [42]. Since *Francisella *has been demonstrated to induce apoptosis in macrophages [43] this might explain, at least in part, the drop in *ripA *expression during the late stage of infection. We are currently investigating the scope and mechanisms of pH associated gene regulation in *Francisella *and its role in host cell adaptation and virulence.

Given that growth media and the stage of infection had similar affects on *iglA *and *ripA *expression we thought it reasonable to determine if the two genes were subject to the same or overlapping regulatory mechanism(s). Earlier microarray and proteomic [22-25] analyses revealed that the expression of *iglA *and IglA, respectively, as well as a number of other genes and proteins, are regulated by two related transcriptional regulators, MglA and SspA [23,44].

Transcriptional profiling studies of *mglA *and *sspA *mutant strains by microarray [23] gave no indication that either of these regulators affected *ripA *expression. However, in complementary proteomic studies, RipA (FTN_0157) was present in 2 - fold higher amounts in a *F. novicida mglA *mutant strain as compared to wild type [25]. This result suggested that MglA has a direct or indirect repressive effect on RipA expression. Our analysis using *ripA'-lacZ *fusion reporter strains revealed that *ripA *expression was increased in both *ΔmglA *and *ΔsspA *mutants, and therefore correlated with the proteomics analysis of MglA mediated gene regulation. Thus, MglA and SspA positively affect *iglA*, but have a negative effect on *ripA *expression *in vitro*. If the intracellular regulation of *iglA *does indeed occur through the activities of MglA and SspA it is likely that in the early stages of *F. tularensis *intracellular replication, the increase in *ripA *expression is mediated by a mechanism that is independent of, or ancillary to, the MglA/SspA regulon.

## Conclusion

Studies focusing on intracellular gene expression are an important aspect of discerning *Francisella *pathogenesis mechanisms. We found that *ripA*, which encodes a cytoplasmic membrane protein that is required for replication within the host cell cytoplasm, is transcribed independently of neighbouring genes. Further, *ripA *is differentially expressed in response to pH and during the course intracellular infection. The intracellular expression pattern of *ripA *mirrored that of *iglA *and other *Francisella *virulence - associated genes that are regulated by MglA and SspA. However, in the transcriptional regulator *deletion *mutants, there were opposing effects on *iglA *and *ripA *expression *in vitro*. Since *ripA *is essentially repressed by MglA and SspA, the increase in *ripA *expression that corresponds with increased MglA/SspA activity *in vivo *suggests that this gene is responsive to an as-of-yet unknown complementary regulatory pathway in *Francisella*.

## Methods

### Bacterial strains and cell culture

*F. tularensis *Live Vaccine Strain (LVS) (Table 1) was propagated on chocolate agar (25 g BHI l^-1^, 10 μg hemoglobin ml^-1^, 15 g agarose l^-1^) supplemented with 1% IsoVitaleX (Becton-Dickson), BHI broth (37 g BHI l^-1^, 1% IsoVitalex), or Chamberlains defined media [26]. All bacterial strains cultured on chocolate agar were grown at 37°C. Broth cultures were incubated in a shaking water bath at 37°C. J774A.1 (ATCC TIB-67) reticulum cell sarcoma mouse macrophage-like cells were cultured in DMEM plus 4 mM L-glutamine, 4500 mg glucose l^-1^, 1 mM sodium pyruvate, 1500 mg sodium bicarbonate l^-1^, and 10% FBS at 37°C and 5% CO_2 _atmosphere.

### Reverse transcriptase PCR

Total RNA was isolated from mid exponential phase cultures using a mirVana RNA isolation kit (Ambion) and procedures. DNA was removed by incubation with RQ1 DNase (Promega) for 1 hour at 37°C. First strand cDNA was generated using SuperScript III Reverse transcriptase (Invitrogen) and random primers. cDNA was quantified using a ND-1000 spectrophotometer (Nanodrop). PCR analysis of *ripA *and *tul4 *expression was accomplished using 20 ng cDNA per 50 μl PCR reaction. As a control for DNA contamination, a Reverse transcriptase reaction was conducted without the Reverse transcriptase enzyme. Ten percent of each reaction was analyzed by agarose gel electrophoresis, ethidium bromide staining, and densitometry using BioRad Quantity One software. Trace intensity (Int mm) of *ripA *was normalized to the mean *tul4 *expression [23]. Mean normalized expression and standard deviation were calculated based on RT-PCR of four samples of RNA derived from independent cultures. Significance was determined using an unpaired two tailed t test with unequal variance.

### Agarose formaldehyde electrophoresis and Northern analysis

Total RNA was harvested from mid exponential phase *F. tularensis *LVS grown in Chamberlains defined media using RNAeasy columns (Qiagen), concentrated by ethanol/sodium acetate precipitation and quantified with a ND-1000 spectrophotometer (Nanodrop). RNA was separated using agarose-formaldehyde (2% agarose, 2.2 M Formaldehyde) electrophoresis followed by capillary transfer to nitrocellulose as described [45]. Additional lanes of the membrane containing duplicate samples were stained with methylene blue to assess rRNA bands for degradation and equality of loading. Digoxigenin labeled RNA probes were generated using a Northern Starter Kit (Roche). Probe generation, hybridization, washing, and detection were performed using the manufacturer's (Roche) protocols.

### Reporter fusion construction and mutagenesis

Specific *F. tularensis *LVS DNA fragments were produced by PCR amplification of genomic DNA using Pfu turbo DNA polymerase (Stratagene). Three DNA fragments were PCR amplified, cloned, and the DNA sequenced for conformity to the published *F. tularensis *LVS DNA sequence. (1) 1300 bp amplicon (primers TTTGGTGTGTTTATCGGTCTTGAAGGCGGTATTGATG and CACGATATCCATTTTATTCCTTTCTAATCCATTTATCC) for the generation of the in-frame *ripA'-lacZ1 *translational fusion of the *ripA *start codon to *lacZ *[46]. (2) 1000 bp amplicon (primers atagcggccgccaggtaaagtgactaaagtacaagataatggtgc and gcgttaattaacctttctaatccatttatccaaaagaatttacac) for the generation of the *ripA'-lacZ2 *transcriptional fusion. (3) 740 bp amplicon (primers agttGCGGCCGCtattccaaccagtgcatttttcactttagtg and TTCCttaattaaCTTATTGTCCTTTTTTTCACAACACCTTATAAGC) for the generation of the *iglA'-lacZ *transcriptional fusion. The *lacZ *reporter vectors pALH109 and pALH122 were used as the source of the translational and gene transcriptional *lacZ *fusion constructs [46]. The translational gene fusion (pALH109) was ligated with a pBSK vector containing the cat gene driven by the *F. tularensis groEL *promoter to construct pBSK *lacZ cat*. The transcriptional gene fusion (pALH122) was ligated with a pBSK vector containing the *aphA1 *allele driven by the *F. tularensis groEL *promoter to construct pBSK *lacZ aphA1*. A *KpnI/EcoRV *fragment containing the *ripA *promoter was ligated to a *SmaI/KpnI *fragment of pBSK *lacZ cat *to form pBSK *ripA'-lacZ1. NotI/PacI *fragments of the cloned promoters were ligated to a *NotI/PacI *fragment of pBSK *lacZ aphA1 *to form pBSK *ripA'-lacZ2 *and pBSK *iglA'-lacZ. KpnI/NotI *fragments from pBSK reporters were ligated to *KpnI/NotI *fragments of pKK MCS to construct pKK *ripA'-lacZ1 *and pKK *ripA'-lacZ2*. All plasmids used in these studies are listed in Table 1.

*Francisella *chromosomal and multicopy reporter strains were generated by transformation of pBSK suicide vectors or pKK shuttle vectors containing the fusion constructs into the *F. tularensis *LVS strains as described [47]. Wild type and reporter alleles of each gene are present in the reporter strains. Site directed mutagenesis of pKK *ripA'-lacZ1 *was performed using the Stratagene QuickChange XL kit and the manufacturers protocols. All *ripA *promoter mutations were confirmed by DNA sequence analysis.

### Measuring β-galactosidase activity expressed by intracellular organisms

To determine the activity of *Francisella *promoter *lacZ *fusions in the intracellular environment, intracellular invasion and replication assays were conducted by adding *F. tularensis *LVS strains cultured to mid exponential phase in BHI to J774A.1 monolayers at a multiplicity of infection (MOI) of 100 in 200 μl tissue culture media. Assays were synchronized as described [14,29]. At 15 minutes post inoculation, monolayers were washed 3 times with pre-warmed tissue culture media to remove extracellular bacteria. At 1, 6, and 24 hours post inoculation samples were washed with PBS and scraped into 200 μl PBS. The number of CFU in each sample was determined by serial dilutions and plating on Chocolate agar. One hundred μl of each sample was lysed in 2× lysis buffer (1% NP40, 0.5 M Tris pH 7.4, 5 mM EDTA) and assayed for β-galactosidase activity using the substrate Chlorophenol red-β-D-galactopyranoside (CPRG). Twenty μl of each sample was mixed with 130 μl of CPRG buffer (2 mM CPRG, 25 mM MOPS pH 7.5, 100 mM NaCl, 10 mM MgCl_2_, 50 mM β-mercaptoethanol) and incubated at 37°C until visible color developed. Enzymatic activity was stopped by adding 80 μl of 0.5 M Sodium Carbonate and OD_580 _measured to calculate substrate conversion. Background β-galactosidase activity was determined at each time point using duplicate samples of J774A.1 cells infected with wild type *F. tularensis *LVS. Mean background activity was subtracted from each sample before calculating relative activity. Relative β-galactosidase activity was calculated by normalizing OD_580 _readings with time of development, dilution of sample, and CFU recovered per sample. Data are presented as activity per 10^10 ^bacteria which results in an activity range similar to Miller units. All assays were performed using four wells of infected cells from a 24 well tissue culture plate per time point. Inoculum activities were determined using the same techniques before addition to cell culture in replicates of four.

Significance was calculated using an unpaired two tailed t test assuming unequal variance. P values of less than 0.05 were considered significant.

### Allelic exchange

A *ripA'-TC *fusion was made by Splice Overlap Extension (SOE) PCR [48] using primers designed to insert the tetracysteine (TC) tag sequence with a glycine linker between the last *ripA *codon and the stop codon (Fig. 4b). Deletion constructs made by SOE PCR retained the start and stop codons of *mglA *(fusion of 1^st ^four and last two codons) and *sspA *(fusion of 1^st ^four and last 4 codons) in frame with 0.8 kb of flanking sequence. The constructs were cloned into pMP590 (Table 1) and sequenced to confirm the integrity of the flanking DNA sequence. Allelic exchange was achieved by transformation, selection for plasmid co-integrates, counter selection on sucrose containing media and confirmed via PCR analysis for replacement of the wild type with the deletion mutant allele as described [47]. Each mutation was confirmed by DNA sequence analysis.

### Extracellular β-galactosidase assay

Overnight cultures of *lacZ *reporter strains were diluted 1:10 in Chamberlains defined media and cultured until mid exponential phase (0.2-0.8 OD_600_). β-galactosidase activity was measured as OD_420_using the substrate ONPG (Sigma) as described elsewhere [49]. Relative promoter activity was normalized using OD_600 _of culture, time of development, and cell to buffer ratio (CBR).

Statistical analysis was performed to determine the mean Miller units and standard deviation from three independent cultures and significance calculated using an unpaired two tailed t test with unequal variance.

### SDS-PAGE and FlAsH™ labelling

Proteins were separated by SDS-PAGE. Total protein loaded in each sample was equivalent as determined by a BCA assay (Pierce). FlAsH™ labeling was accomplished using the manufactor's protocols (Invitrogen). In gel fluorescence of the arsenical fluoriscein and total protein stain was conducted on a Typhoon 9200 laser scanner (488 nm laser/520 nm BP 40 filter and 633 nm laser/670 nm BP 30 filter). Densitometry was conducted using ImageQuant XL software and sample comparisons made using the same gel and scan. Mean intensity and standard deviation of four samples from independent cultures was calculated and significance determined using an unpaired two tailed t test with unequal variance.

## Authors' contributions

JF carried out all experiments with the participation of TMK and SB in the extracellular galactosidase assays. TMK and SB helped draft the manuscript and provided intellectual input to data analysis. THK and JF designed and coordinated experiment, analyzed data, and drafted the manuscript. All authors read and approved the final manuscript.
